# Genome sequence data of *Burkholderia* sp. IMCC1007 isolated from maize rhizosphere: A potential strain in fusaric acid mycotoxin biodegradation

**DOI:** 10.1016/j.dib.2023.109204

**Published:** 2023-05-06

**Authors:** Abd Rahman Jabir Mohd Din, Nor Zalina Othman

**Affiliations:** a Innovation Centre in Agritechnology for Advanced Bioprocess (ICA), Universiti Teknologi Malaysia, Pagoh Education Hub, 84600 Muar, Johor, Malaysia; b Faculty of Chemical and Energy Engineering, Universiti Teknologi Malaysia, 81310 Johor Bahru, Malaysia

**Keywords:** *Burkholderia*, Genome sequence, Mycotoxin degradation, Fusaric acid

## Abstract

*Burkholderia* sp. IMCC1007 is a gram-negative, aerobic bacterium affiliated with class Betaproteobacteria, which was successfully isolated from maize rhizospheric soil sample in UTM research plot, Pagoh, Malaysia by using enrichment method. Strain IMCC1007 utilized 50 mgL^−1^ fusaric acid as its carbon source and degraded it completely within 14 h. Genome sequencing was performed using Illumina NovaSeq platform. The assembled genome was annotated using RAST (Rapid Annotation Subsystem Technology) server. The genome size was approximately 8,568,405 base pairs (bp) in 147 contigs with a G+C content of 66.04%. The genome includes 8,733 coding sequences and 68 RNAs. The genome sequence has been deposited at GenBank with the accession number of JAPVQY000000000. In the pairwise genome-to-genome comparisons, the strain IMCC1007 had an average nucleotide identity (ANI) of 91.9% and digital DNA-DNA hybridization (dDDH) value of 55.2% with *Burkholderia anthina* DSM 16086^T^ respectively. Interestingly, fusaric acid resistance gene (*fus*C) and *nic*ABCDFXT gene clusters (hydroxylation of pyridine compound) were found in the genome. Additionally, preliminary genome annotation analysis of strain IMCC1007 identified tryptophan halogenase (*prn*A) gene responsible for antifungal pyrrolnitrin biosynthesis. This dataset herein provides further insights into the fusaric acid degradation mechanism of the genus *Burkholderia*.

**Specifications Table**

**Table:** 

Subject	Microbiology
Specific subject area	Environmental Microbiology
Type of data	Genome assembly, predicted genes and annotation
How the data were acquired	The whole genome sequencing data was determined using the NovaSeq Illumina platform following de novo genomic assembly
Data format	Raw, analysed and assembled genome sequences
Parameters for data collection	The genomic DNA extraction, fragment library preparation, Illumina sequencing, de novo assembly and annotation
Description of data collection	The genomic DNA was extracted, before sequencing using NovaSeq sequencer (Illumina). The reads were quality trimmed. *De novo* assembly of genome sequences was performed using SPAdes version 3.14. Annotation was performed using the RAST server.
Data source location	*Burkholderia* sp. IMCC1007 was isolated from soil samples, following the harvest of maize crops grown under organic management at research farm located on the Universiti Teknologi Malaysia (UTM) (2°15.6406’N, 102° 73.2332’E; Pagoh, Malaysia). The strain IMCC1007 was deposited in the ICA Microbial Culture Collection (IMCC) of UTM.
Data accessibility	The complete genome sequence of *Burkholderia* sp. IMCC1007 was deposited in NCBI GenBank database under accession number JAPVQY000000000 Direct URL to data: https://www.ncbi.nlm.nih.gov/nuccore/JAPVQY000000000 Database link: BioProject: https://www.ncbi.nlm.nih.gov/bioproject/PRJNA910835 Biosamples: https://www.ncbi.nlm.nih.gov/biosample/SAMN32146873

## Value of the Data

• This whole-genome sequence of *Burkholderia* sp. IMCC1007 could provide valuable information related to fusaric acid (mycotoxin) detoxification strategies.
• The genomic information of this strain IMCC1007 would be useful for comparative genomic analysis of other *Burkholderia* strains with biodegradation capability.
• The genome data presented herein can be used by researchers working in the field of biodegradation and bioremediation of mycotoxin and various aromatic toxic compounds.

## 1 Objective

Mycotoxin contamination is a serious threat in agriculture global industry and causes severe human health problems. Effective measure through microbiological approach has been extensively a preferable choice as it offers higher efficiency and more environmentally sustainable. Screening for mycotoxin-degrading bacteria by enrichment strategies is gaining a greater emphasis. To date, only four species were successfully isolated, capable of utilizing fusaric acid as a sole carbon source, namely *Klebsiella oxytoca* strain HY-1, *Pseudomonas cepacia* strain UK1, *Stenotrophomonas maltophilia* strain K27a and *Burkholderia ambifaria* strain T16 [1], [2], [3], [4]. *Burkholderia* is a genus that belongs to the Betaproteobacteria class, widely distributed in diverse ecological niches [5]. *Burkholderia* sp. IMCC1007 has been assessed to be useful as a biodetoxification agent because it completely metabolized fusaric acid (mycotoxin) within shorter time. Here, we present a complete genome sequence of *Burkholderia* sp. IMCC1007 which was isolated from maize rhizospheric soil, for the mechanism elucidation that are responsible for fusaric acid degradation. So far, the fusaric acid degradation genes which potentially to be used in the energy production were rarely discussed. These data further support the potential for applying IMCC1007 for mycotoxin degradation, hence reducing the utilization of chemical fungicides.

## 2 Data Description

**Fig. 1:**
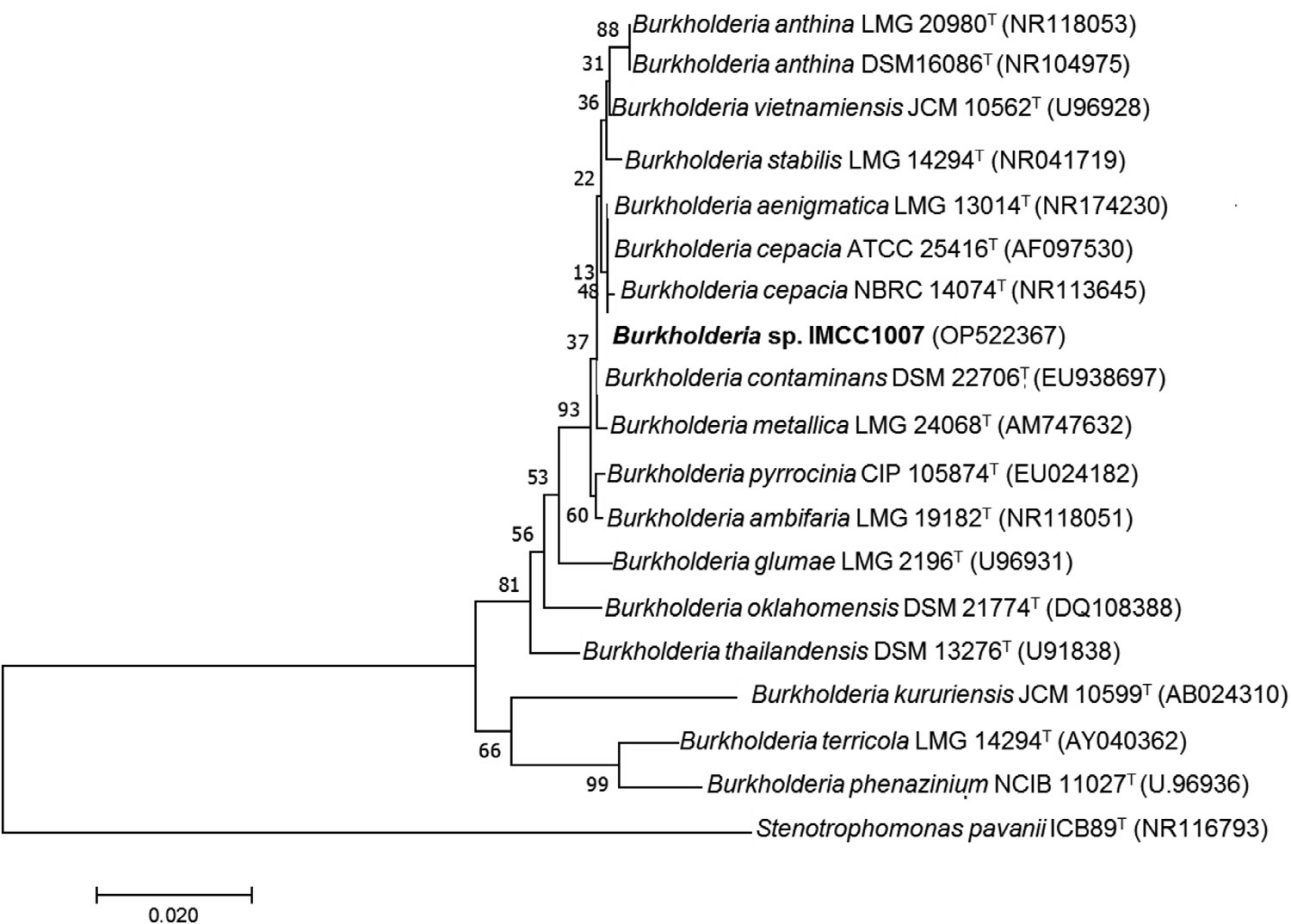
A phylogenetic tree based on 16S rRNA gene sequences created using Neighbor-Joining Method and showing the relationships between *Burkholderia* sp. IMCC1007 with the closed species. The numbers at branch nodes indicated bootstrap percentages derived from 1000 replicates. *Stenotrophomonas pavanii* ICB89^T^ was used as the outgroup.

*Burkholderia* sp. IMCC1007 was isolated from soil sample during the maize core rhizosphere microbial diversity investigation at research farm located at Universiti Teknologi Malaysia, Pagoh, Malaysia. This study presents the complete genome sequence of *Burkholderia* sp. IMCC1007. The genome sequencing was performed using the NovaSeq Illumina platform. Quality-filtered sequences were assembled using SPAdes Genome Assembler (v.3.14). The assembled genome was annotated using the RAST server. The result showed that the strain IMCC1007 genome contained 8,568,495 base pairs (bp) and N50 value of 99,399 with 66.01% G + C content respectively. *Burkholderia* sp. IMCC1007 was deposited at GenBank under the accession number JAPVQY000000000, and consists of sequences JAPVQY010000001 - JAPVQY010000138 (BioProject ID: PRJNA910835 and BioSample ID: SAMN32146873). The genome features of strain IMCC1007 were summarized in Table 1. Fig. 1 showed that *Burkholderia* sp. IMCC1007 was closely related to several type strains of *Burkholderia cepacia* and formed a clade with *Burkholderia cepacia* NBRC 14074^T^ with relatively high similarity (99%). BLAST analysis of the 16S rRNA gene sequences (1408bp) of strain IMCC1007 was performed, where significant groupings were downloaded. The 16S rRNA gene sequence of this strain was deposited in the GenBank database with accession number OP522367. Another relevant phylogenetic marker would be needed to resolve the species discrimination that were indistinguishable by determining the utility of *recA* and *gyrB* gene sequencing, which provides higher resolution than 16S rRNA sequence analysis. Although phylogenetic analysis of 16S RNA gene relatedness demonstrated high similarity, an analysis between strain IMCC1007 and reference strains using average nucleotide identity (ANI) and digital DNA-DNA hybridization (dDDH) was employed to predict further taxonomic affiliation at the genomic level. The strain IMCC1007 demonstrated ANI values of 91.90% and 89.06% with *B. anthina* DSM 16086^T^ and *B. ambifaria* T16, respectively. This similar trend was consecutively followed by the dDDH values between *Burkholderia* sp. IMCC1007 and *B. anthina* DSM 16086^T^, which exhibited the highest percentage (55.2%) albeit lower in values, succeeded by *B. ambifaria* T16, with a dDDH value of 49.8% out of 17 strains. From Table 2, the ANI and dDDH values for strain IMCC1007 were below the recommended threshold values for species delineation (95-96% for ANI and 70% for dDDH). Hence, we proposed *Burkholderia* sp. IMCC1007 as an undescribed species member of *Burkholderia* due to its distinguishable from others.

**Table 1:** Genome characteristics of *Burkholderia* sp. IMCC1007.

Features	Strain IMCC1007
Total sequence length (bp)	8,568,495
Number of contigs	138
N50	99399
L50	24
G + C content (%)	66.01
Number of coding sequences (CDS)	8733
RNAs	68
Number of subsystems	394
NCBI accession number	JAPVQY000000000

**Table 2:** Taxonomic affiliation of strain IMCC1007 based on average nucleotide identity (ANI) and digital DNA-DNA hybridization (dDDH).

No.	Reference strains	Size (bp)	ANIb values (%)	dDDH value (%)
1.	*Burkholderia cepacia* NBRC 14074^T^	8,486,058	87.74	45.9
2.	*Burkholderia cepacia* ATCC 17759^T^	8,558,034	87.97	48.7
3.	*Burkholderia cepacia* ATCC 25416^T^	8,366,370	87.70	46.1
4.	*Burkholderia aenigmatica* LMG 13014^T^	9,000,646	87.55	43.0
5.	*Burkholderia contaminans* LMG 23361^T^	9,263,236	87.65	42.2
6.	*Burkholderia gladioli* IHBB13602	8,151,847	78.39	21.8
7.	*Burkholderia pseudomallei* CM 000113	7,300,914	80.43	24.7
8.	*Burkholderia pyrrocinia* DSM 10685^T^	7,847,862	88.00	40.0
9.	*Burkholderia ambifaria* T16	7,358,950	89.06	49.8
10.	*Burkholderia anthina* DSM 16086^T^	7,623,315	91.90	55.2
11.	*Burkholderia diffusa* DSM 23434^T^	7,096,255	88.75	42.3
12.	*Burkholderia plantarii* ATCC 43733^T^	7,883,689	78.97	22.7
13.	*Burkholderia oklahomensis* C6786	7,135,022	80.65	25.6
14.	*Burkholderia terricola* LMG 10929^T^	7,321,401	76.42	18.8
15.	*Burkholderia ubonensis* ATCC 31433^T^	7,687,481	85.02	35.4
16.	*Burkholderia glumae* ATCC 33617^T^	6,480,185	78.65	22.7
17.	*Burkholderia vietnamiensis* LMG 10929^T^	6,827,876	86.56	41.5

All strains with superscript capital T at the end of accession numbers used for comparison were the type strains

**Fig. 2:**
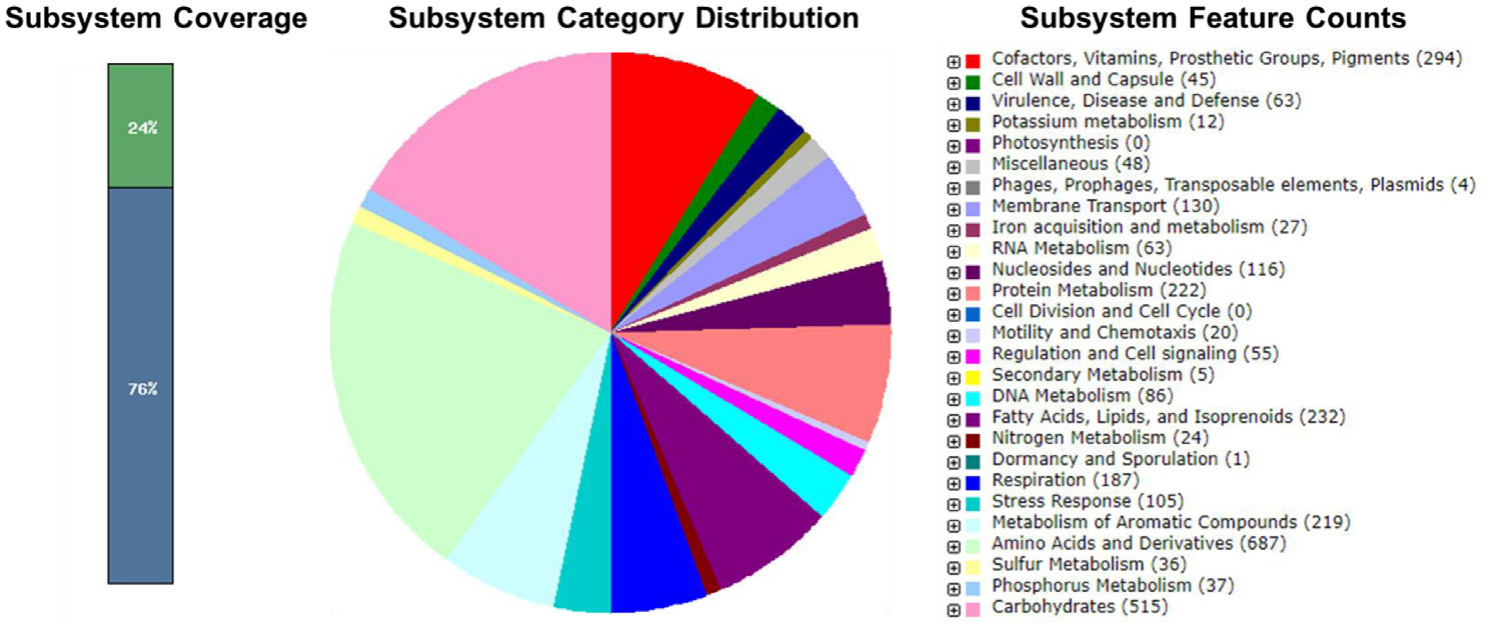
Subsystem statistics information of *Burkholderia* sp. IMCC1007 using RAST annotation.

The genome of *Burkholderia* sp. IMCC1007 consisted 138 contigs, containing 8,733 coding sequences and 68 RNAs with 21% subsystem coverage. Based on the RAST annotation, the most abundant subsystem feature was metabolism of amino acid derivatives (*n* = 687 CDSs), followed by carbohydrates (*n* = 515 CDSs), vitamin and protein metabolisms with 294 and 222 CDSs respectively. Besides primary metabolism, 43 CDSs and 105 CDSs were assigned to the genes encoding for resistance to antibiotic and toxic compounds as well as detoxification respectively (Fig. 2).

Preliminary annotation analysis revealed key genes associated with fusaric acid detoxification, contained a *fus* operon (*fus*ACE, fusaric acid resistance genes). The *fus*ACE proteins of strain IMCC1007 showed 75.5%, 89.2% and 87% protein similarities to proteins of *Burkholderia cepacia* respectively (Table 3). Tryptophan halogenase gene (*prn*A) and presqualene diphosphate synthase (*hpn*D) encoding for biosynthesis of pyrrolnitrin and terpene were also detected in the genome sequence [6]. Notably, a subset of the phenazine biosynthesis genes was also identified with high correlation values [7]. Specialized antimicrobial metabolite, fragin biosynthetic gene cluster was present within this bacterial genome, as described in *Burkholderia cenocepacia* H111 [8]. Table 3 summarized the promising antibiotic biosynthetic genetic diversity encoded by this strain. Of note, this genome strain has gene clusters (*nic*ABCDFXT) encoding enzymes for carboxylic derivative of pyridine compounds degradation that linking to maleamate and tricarboxylic acid (TCA) oxidative pathways through fumaric acid intermediate [9,10]. Possibly, this metabolism process was contributed to fusaric acid (a pyridine derivative) degradation in the energy production of strain IMCC1007. Aerobic degradation of some of *N*-heterocyclic aromatic toxic compounds of major environmental concerns including nicotinic acid, picolinic acid and 2,5-dihydroxypyridine have been reported so far in different bacterial species [11,12]. Thus, bacteria with *fus*C and *nic*ABCDFXT gene clusters including strain IMCC1007 might be important in fungal toxin fusaric acid degradation.

**Table 3:** Responsible genes found in the strain IMCC1007 genome.

Gene name	Predicted functions	Strains	Identity (%)
*fus*A	Fusaric acid resistance protein A	*Burkholderia cepacia* ATCC 25416^T^	75.5
*fus*C	Fusaric acid resistance protein C	*Burkholderia cepacia* ATCC 25416^T^	89.2
*fus*E	Fusaric acid resistance protein E	*Burkholderia cepacia* ATCC 25416^T^	87
*nic*A	Nicotinate dehydrogenase subunit A	*Pseudomonas putida* DSM 6125^T^	67.1
*nic*B	Nicotinate dehydrogenase subunit B	*Pseudomonas putida* DSM 6125^T^	58.4
*nic*C	6-hydroxynicotinate monooxygenase	*Bordetella bronchiseptica* NCTC13252^T^	71.4
*nic*D	*N*-formylmaleamate deformylase	*Pseudomonas putida* DSM 6125^T^	57.7
*nic*F	Maleamate amidohydrolase	*Pseudomonas putida* DSM 6125^T^	42.2
*nic*X	2,5-dihydroxypyridine 5,6-dioxygenase	*Pseudomonas putida* DSM 6125^T^	54.7
*nic*T	Putative metabolite transport protein	*Pseudomonas putida* DSM 6125^T^	58.7
*mai*A	Maleate isomerase	*Pseudomonas putida* DSM 6125^T^	76.8
*prn*A	Tryptophan halogenase	*Burkholderia* sp. IDO3	95.2
*hpnD*	Presqualene diphosphate synthase	*Rhodopseudomonas palustris* CGA009	63.4
*Gra*	Granaticin polyketide synthase	*Streptomyces violaceoruber* ATCC 14980^T^	50.4
*phzE*	Anthranilate synthase component I	*Pseudomonas aeruginosa* ATCC 15692^T^	99.8
*phzF*	Phenazine biosynthesis isomerase	*Burkholderia anthina* LMG 20980^T^	94.2
*hamE*	Polyketide cyclase	*Burkholderia cenocepacia* H111	94.4
*hamG*	Aminotransferase class III	*Burkholderia cenocepacia* H111	97.7

All strains with superscript capital T at the end of accession numbers used for comparison were the type strains

## 3 Experimental Design, Materials and Methods

### 3.1 Sample collection and bacterial isolation

A fusaric acid-degrading bacteria was isolated from rhizosphere maize soil sample from Pagoh, Malaysia by an enrichment method. 5 g of the collected soil sample was suspended into 45 mL minimal medium supplemented with 50 mgL^−1^ of fusaric acid as a sole carbon and energy source at 30°C. After serial enrichments, the diluted culture was spread on minimal media containing fusaric acid for 5 days. One bacterial strain designated as IMCC1007 was selected due to its potential to degrade and detoxify fusaric acid completely within 14 h. This strain was deposited in the ICA Microbial Collection Center (IMCC) and preserved in 50% glycerol stock at -80°C.

### 3.2 DNA extraction

Strain IMCC1007 was grown in 20 mL of 1/10 TSB medium at 30°C for 24h at 150 rpm. After overnight culture, genomic DNA of strain IMCC1007 was extracted using phenol-chloroform standard method. Then, an upper aqueous layer was precipitated by adding 1/10 volume of 3 M sodium acetate (pH 5.2) and equal volume of 100% 2-propanol before overnight incubation at -20°C. Pellet was collected after centrifugation and washed with 70% and 100% ethanol consecutively. The extracted DNA sample was dried and dissolved in 50 μL of nuclease-free water. The quantity and quality of the purified genomic DNA of strain IMCC1007 were then verified using Qubit fluorometer (Thermo Fisher Scientific, USA). An extracted DNA was confirmed using 1% agarose gel by electrophoresis with appearance of 10 kbp clear band without smear, indicating no RNA contamination.

### 3.3 Illumina library construction, genome sequencing and annotation

Sequencing library was prepared with a 350-bp using NEB Ultra II library preparation kit according to the manufacturer protocol (NEB, Ipswich, MA). Library was sequenced on a NovaSeq 6000 platform (Illumina, San Diego, CA) with a genome coverage of 99.98%. The whole genome of strain IMCC1007 was assembled via SPAdes (v.3.14) [13]. Sequence adaptors and low quality reads were filtered using Unicycler (v.0.4.8) [14]. The complete genome annotation was carried out using the RAST server (http://www.rast.nmpdr.org) [15] and gene functions were annotated based on KEGG (Kyoto Encyclopedia of Genes and Genomes) database [16].

### 3.4 16S rRNA gene sequence and phylogenetic analysis

The 16S rRNA gene sequence of strain IMCC1007 (1408 bp) was analyzed following BLAST search retrieved from GenBank database of reference sequences. All sequences were aligned with ClustalW and phylogenetic tree was constructed by using Neighbor-Joining method with bootstrap analysis (1000 replicates) in MEGA 7.0 software package [17].

Average nucleotide identity and digital DNA-DNA hybridization

JSpeciesWS algorithm (https://jspecies.ribohost.com/jspeciesws/) was used to compute mean nucleotide identity of strain IMCC1007 with reference genomes [18] and digital DNA-DNA hybridization (dDDH) values were performed using the Type Strain Genome Server (TYGS) (https://tygs.dsmz.de/) [19].

## Ethics Statements

NA

## CRediT authorship contribution statement

**Abd Rahman Jabir Mohd Din:** Conceptualization, Methodology, Writing – original draft, Writing – review & editing, Data curation, Project administration. **Nor Zalina Othman:** Writing – review & editing, Validation, Supervision.

## Declaration of Competing Interest

The authors declare that they have no known competing financial interests or personal relationships that could have appeared to influence the work reported in this paper.

## Data Availability

Burkholderia sp. IMCC1007, whole genome shotgun sequencing project (Original data) (NCBI).
